# Morel-Lavallée Lesion of the Hip: A Case Report

**DOI:** 10.7759/cureus.93069

**Published:** 2025-09-23

**Authors:** Abhineet Dey, Piyal Nag

**Affiliations:** 1 Radiology, Silchar Medical College and Hospital (SMCH), Silchar, IND; 2 Radiology, Silchar Cancer Centre, Silchar, IND

**Keywords:** ct, imaging, internal degloving, morel-lavallée lesion, mr, radiology, surgery, trauma, usg

## Abstract

Morel-Lavallée lesion (MLL) is a rare post-traumatic internal degloving injury caused by tangential shear forces separating subcutaneous tissue from underlying fascia. We report a 29-year-old male patient with a progressive, fluctuant swelling over the right hip and thigh following a fall. Ultrasound revealed an anechoic fluid collection in the subcutaneous plane of the hip, which yielded serosanguinous fluid on aspiration. CT confirmed the extent of the lesion over the hip and thigh, while MRI further characterized the collection as an acute lesion without any pseudocapsule formation. Surgical drainage with pressure dressing led to complete recovery. MLL should be considered a key differential in post-traumatic soft tissue swellings, especially in the characteristic locations. A multimodal imaging approach can be helpful to characterize and stage the lesion for proper management.

## Introduction

Morel-Lavallée lesion (MLL) is a rare traumatic closed-type of internal degloving injury resulting in separation of superficial and deep fascia. Tangential shear forces rupture the integumentary hemolymphatic channels, causing fluid accumulation between the separated layers [1-3]. Although uncommon, MLL should be considered a key differential in post-traumatic soft tissue swellings, especially around the pelvic region [4]. Early recognition is crucial, as delayed diagnosis increases the risk of chronic pseudocapsule formation, recurrence, and treatment failure [5].

We report the case of a 29-year-old man who developed progressive swelling over the right hip and thigh after a fall. Initial ultrasound identified an anechoic, yielding a serosanguinous fluid on aspiration. CT and MR further characterized the extent and chronicity of the lesion. Given the typical history and characteristic location over the hip and thigh, a diagnosis of MLL was made. The collection was subsequently managed during hospital stay with surgical incision and drainage followed by post-operative pressure dressing.

This case highlights the value of a stepwise imaging approach, the role of ultrasound as a first-line tool, and practical considerations for management. It also underscores the importance of maintaining a high index of suspicion for MLL in post-traumatic soft tissue swellings to ensure timely and effective treatment.

This case was presented as a poster presentation at the 23rd Asian Oceanian Congress of Radiology (AOCR) and the 77th Annual Conference of Indian Radiological & Imaging Association (IRIA) on January 24th, 2025.

## Case presentation

**Figure 1:**
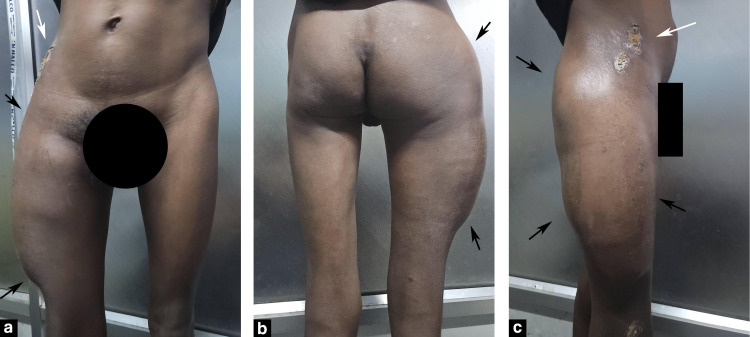
(a-c) Clinical examination revealed a non-tender broad-based fluctuant swelling over the right greater trochanter region extending to the knee and the hip (black arrows). Note the old healed abrasion over the previous impact site (a) & (c) (white arrow). Black arrows indicate the extent of the swelling. The white arrow shows the old-healed abrasion at the impact site.

The patient, a 29-year-old man, presented to the emergency room with a large, fluctuant non-tender swelling over his right hip and thigh (Figure 1). The patient had a history of fall from his bicycle over a pile of bricks five days earlier, following which he started to develop swelling in the region of the thigh after 36 hours. The swelling progressed in size above the hip over the course of three days, following which he presented to us. The patient was completely ambulatory with stable vitals.

**Figure 2:**
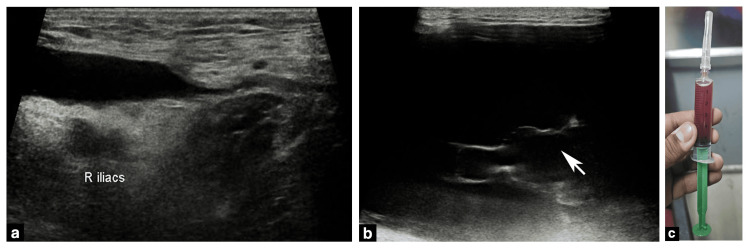
(a, b) Emergent ultrasound (US) examination revealed a large anechoic fluid collection in the subcutaneous plane of the right hip with (b) incomplete internal septations (white arrows). (c) US-guided aspiration yielded a serosanguinous fluid. (a) Right iliac vessels outline the medial border of the collection. (b) White arrow points to incomplete internal septations within the collection

**Figure 3:**
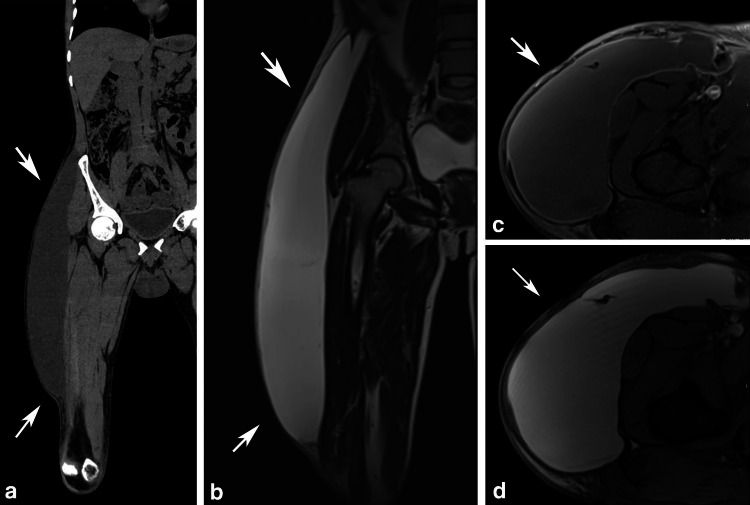
Cross-sectional imaging using non-contrast CT (a) & MRI (b and c) confirmed the characteristic subcutaneous location and extent of the lesion. The collection demonstrated intermediate signal on T2-weighted images (b and d) and intermediate signal with a thin partial capsular enhancement in the T1-weighted post-contrast image (c) (a) Coronal non-contrast CT image of the thigh and hip, (b) Coronal T2-weighted MRI of the thigh and hip, (c) Axial post-contrast fast-suppressed T1-weighted MRI of the thigh, and (d) Axial T2-weighted MRI of the thigh White arrows indicate the extent of the collection. CT: Computed tomography, MRI: Magnetic resonance imaging

Emergent ultrasound (US) examination demonstrated a large anechoic fluid collection between the superficial and deep fascia of the subcutaneous tissue of the involved region. US-guided aspiration yielded a serosanguinous fluid (Figure 2). Cross-sectional CT and MR imaging demonstrated the complete extent of the collection and contrast-enhanced magnetic resonance studies further demonstrated intermediate T1 & high T2 signal characteristics with no evidence of capsule formation consistent with the serosanguinous nature of the fluid aspirated earlier (Figure 3). A diagnosis of MLL was made from the characteristic location of the collection in the context of tangential injury over the hip region.

**Figure 4:**
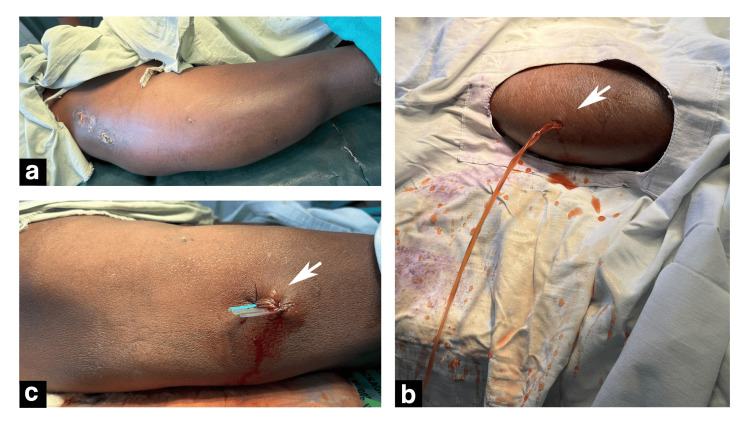
(a-c) Percutaneous incision and drainage of the swelling. (b) A cruciate incision was made on the most dependent part of the collection from which the collection was evacuated (white arrow). (c) A corrugated sheet drain was placed in situ via the incision site (white arrow). (a) Intra-operative presentation before incision. White arrows point to the incision and drainage site (b) and the corrugated sheet drain placed in situ (c).

The case underwent a percutaneous drainage via a cruciate incision followed by pressure bandaging with a corrugated rubber drain in situ. Pressure bandaging was applied over the entire collection area (Figure 4). The patient was discharged after 72 hours of hospital stay following drain removal and advised to wear continued compression garment for at least a month. 

## Discussion

MLL is a rare form of closed internal degloving injury in which tangential impact forces result in a traumatic separation of the skin & subcutaneous tissue from the underlying fascia [1-3]. The lesion most commonly occurs around the greater trochanter region, with an incidence of up to 8.3% in patients with pelvic trauma in some series [3,4].

**Figure 5:**
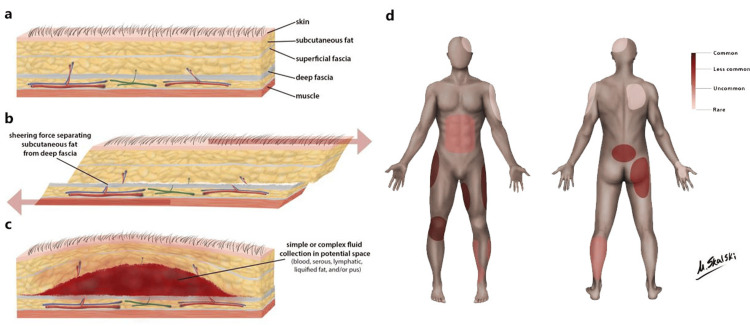
(a–c) Diagrammatic cross-sections from skin to bone showing the tissue changes in a Morel-Lavallée lesion. a: Normal anatomy of skin, subcutaneous tissue, fascia, and bone. b: Shearing forces separate the superficial and deep fascial layers. c: Vascular disruption leads to hemolymphatic fluid accumulation within the potential space. d: Illustration of body regions reported to be affected by Morel-Lavallée lesions, with the pelvis and lower extremities being the most commonly involved sites. Reproduced from [8] under Creative Commons CC-NC-BY-SA 3.0 license.

The shearing type of injury causes disruption of the perforating venolymphatic structures across the subcutaneous plane, resulting in the characteristic fluid collection between the affected planes [6]. This appears as a large flat or fusiform collection between the deep fat subcutaneous fat and the overlying fascia on imaging (Figure 5) [7].

Most cases present at the acute stage, where management is straightforward and recurrence risk is low. As the lesion evolves, partial organization of blood products occurs, followed by the eventual development of a fibrous pseudocapsule surrounding the collection in later stages [5]. Long-standing cases carry a significantly higher risk of recurrence, even after repeated drainage, due to the presence of this fibrous pseudocapsule, which impedes reabsorption of the contained fluid [9,10]. Chronic cases can benefit from further characterization with MRI, as it not only stages the collection but also confirms pseudocapsule formation, thereby guiding appropriate management. Nonetheless, the majority of cases are diagnosed clinically, requiring imaging for only basic characterization of the collection, and such advanced imaging evaluation is not usually warranted.

In our case, the fluctuant nature and characteristic location of the swelling, along with the recent history of trauma, were sufficient to establish the diagnosis, and ultrasound adequately confirmed the findings. Additional imaging was not strictly necessary; however, CT and MRI were performed for academic interest and provided complementary information. CT delineated the full extent of the collection, while MRI demonstrated intermediate T1 and high T2 signal characteristics with mild fluid layering and no evidence of pseudocapsule formation, findings consistent with an acute-to-subacute stage. 

Given these imaging features, it was essential to distinguish the lesion from other potential post-traumatic or infective conditions. Our case was initially referred to as a post-traumatic psoas abscess; however, this diagnosis was excluded based on negative clinical findings, as the patient was afebrile and presented with a superficial, non-tender, fluctuant swelling. Other infective causes, such as intramuscular abscess, were similarly ruled out due to the absence of systemic or local inflammatory signs. An intramuscular hematoma is another possible post-traumatic entity, but these usually arise in the context of more severe trauma, are often associated with underlying fractures, and typically demonstrate a deeper intramuscular location of fluid collection, features not present in our case [11,12]. Bursitis was also excluded, as the collection was superficial and not confined to a recognized bursal sac [13].

Aspiration and intraoperative findings confirmed a serosanguinous collection without a pseudocapsule, consistent with an acute-to-subacute stage. Despite the absence of encapsulation, the lesion’s size and symptoms made conservative management unsuitable, and surgical drainage was performed to ensure resolution and prevent recurrence.

## Conclusions

MLL is a post-traumatic internal degloving injury that should be suspected in patients presenting with fluctuant subcutaneous swellings following tangential trauma. Diagnosis is primarily clinical, supported by imaging evidence of subcutaneous fluid collection, while MRI may be useful in selected cases to assess chronicity and pseudocapsule formation with implications for treatment. In the present case, a diagnosis of a subacute MLL was established after excluding other causes of thigh swelling, such as psoas abscess, intramuscular abscess, and hematoma. The patient was successfully managed with incision and drainage followed by postoperative compression dressing, underscoring the importance of early recognition, accurate staging, and timely intervention to prevent recurrence.
